# In Silico Evaluation of Binding of 2-Deoxy-D-Glucose with Mpro of nCoV to Combat COVID-19

**DOI:** 10.3390/pharmaceutics14010135

**Published:** 2022-01-06

**Authors:** Anirudh Pratap Singh Raman, Kamlesh Kumari, Pallavi Jain, Vijay Kumar Vishvakarma, Ajay Kumar, Neha Kaushik, Eun Ha Choi, Nagendra Kumar Kaushik, Prashant Singh

**Affiliations:** 1 Department of Chemistry, Atma Ram Sanatan Dharma College, University of Delhi, New Delhi 110021, India; as3597@srmist.edu.in (A.P.S.R.); vijayitsbbd@gmail.com (V.K.V.); 2 Department of Chemistry, Sri Ramasami Memorial (SRM) Institute of Science and Technology, Modinagar, Ghaziabad 231206, India; Pallavij@srmist.edu.in; 3 Department of Zoology, Deen Dayal Upadhyaya College, University of Delhi, New Delhi 110078, India; kamleshkumari@ddu.du.ac.in; 4 Department of Chemistry, Indian Institute of Technology, New Delhi 110016, India; ajaykumar@iitd.ac.in; 5 Department of Biotechnology, The University of Suwon, Hwaseong 18323, Korea; neha.bioplasma@suwon.ac.kr; 6 Plasma Bioscience Research Center, Department of Electrical and Biological Physics, Kwangwoon University, Seoul 01897, Korea; ehchoi@kw.ac.kr

**Keywords:** 2-deoxy-D-glucose, main protease of SARS-CoV-2, molecular docking, molecular dynamics simulations, density functional theory calculations

## Abstract

COVID-19 has threatened the existence of humanity andthis infection occurs due to SARS-CoV-2 or novel coronavirus, was first reported in Wuhan, China. Therefore, there is a need to find a promising drug to cure the people suffering from the infection. The second wave of this viral infection was shaking the world in the first half of 2021. Drugs Controllers of India has allowed the emergency use of 2-deoxy-D-glucose (2DG) in 2021 for patients suffering from this viral infection. The potentiality of 2-deoxy-D-glucose to intervene in D-glucose metabolism exists and energy deprivation is an effective parameter to inhibit cancer cell development. Once 2DG arrives in the cells, it becomes phosphorylated to 2-deoxy-D-glucose-6-phosphate (2-DG6P), a charged molecule expressively captured inside the cells. On the other hand, 2DG lacks the ability to convert into fructose-6-phosphate, resulting in a hampering of the activity of both glucose-6-phosphate isomerase and hexokinase, and finally causing cell death. Hence, the potential and effectiveness of 2DG with the main protease (Mpro) of novel coronavirus (nCoV) should be investigated using the molecular docking and molecular dynamics (MD) simulations. The ability of 2DG to inhibit the Mpro of nCoV is compared with 2-deoxyglucose (2DAG), an acyclic molecule, and 2-deoxy-D-ribose (2DR). The binding energy of the molecules with the Mpro of nCoV is calculated using molecular docking and superimposed analysis data is obtained. The binding energy of 2DG, 2DR and 2DAG was −2.40, −2.22 and −2.88 kcal/mol respectively. Although the molecular docking does not provide reliable information, therefore, the binding affinity can be confirmed by molecular dynamics simulations. Various trajectories such as Rg, RMSD, RMSF, and hydrogen bonds are obtained from the molecular dynamics (MD) simulations. 2DG was found to be a better inhibitor than the 2DAG and 2DR based on the results obtained from the MD simulations at 300 K. Furthermore, temperature-dependent MD simulations of the Mpro of nCoV with promising 2DG was performed at 295, 310 and 315 K, and the effective binding with the Mpro of nCoV occurred at 295 K. With the use of DFT calculations, optimized geometry and localization of electron density of the frontier molecular orbitals were calculated.

## 1. Introduction

SARS-CoV-2 has recently received much attention around the world and has been proclaimed a global health emergency. Infection due to novel coronavirus was first reported in Wuhan, China, in December 2019. The genome has a spherical or pleomorphic form that is encased in a glycoprotein structure on its surface [1,2,3]. As COVID-19 cases keep rising exponentially during the waves (1st and 2nd), researchers are working to test antivirals and other therapies in parallel to other interventions [4]. Till date, there is currently no promising and cost-effective medications to treat COVID-19 infection. Researchers are currently working on the development of therapeutic medications to cure coronavirus infections and bring life back to normal [5,6,7]. Remdesivir, hydroxychloroquine and many more options have been reported to be promising molecules against the viral infection [8,9,10,11,12,13,14,15]. Several mutations in the SARS-CoV-2 have been reported and found to be more infectious than previous ones. The infections due to the mutated virus have shaken the world. On 8 May 2021, the Drugs Controller General of India (DCGI) gave the green light for the emergency use of 2-deoxy-D-glucose (2DG) against the infection due to the SARS-CoV-2. Several trials by the Deference Research and Development Organization (DRDO) and Dr. Reddy’s Laboratory, India, were made in several hospitals across India. They found 2DG to be a safe and promising drug for patients with COVID-19. 2DG was administered to patients and produced a positive response; it became independent of additional oxygen. 2DG preserves healthy cells by lowering fixation operations. 2DG is being administered because it does not involve in glycolysis and inhibits the phosphorylation of glucose to produce glucose-6-phosphate. The function of hexokinase-2 (HK2) in connecting ATP from oxidative phosphorylation to the rate-limiting step of glycolysis may aid tumor cell growth. Inhibiting HK2 has been shown to boost the efficacy of anti-cancerous medication and sensitive resistant cells. It plays an important role as a marker in glucose uptake and hexokinase activity [16,17]. Generally, tumor cells have the ability to take more glucose, and therefore to take 2DG in large amounts, hampering the growth of cancer or tumor cells [18,19,20]. Therefore, it may be considered a safe molecule mainly for people below 40 years and the patients does not requite additional oxygen [16,21,22,23,24,25]. Localization of electron density on 2DG can be determined by performing density functional theory (DFT) calculations, and it can predict the electrophilic and nucleophilic sites. DFT calculations are also used to obtain information on the electronic structure of the molecule, as well as the energies and spectroscopic data, including NMR, IR and UV spectra [26,27]. The binding efficiency of molecule and ligands can be studied with molecular docking. PARDOCK is a widely accepted computational tool used in molecular docking to predict the binding affinity between the proteases and ligands. The process is to ascertain about the set of molecules could favorably fit in the binding pocket of protease/receptor or not. Additionally, there is a possibility of forming various interactions such as hydrogen bonds between the protease and ligand [28]. Molecular dynamics (MD) simulation is based on numerical solving equations; it is expressed with physical laws that involve molecular interactions. One of the most popular and accepted tools for MD simulations is GROMACS for better understanding of the formation of the complex between the protease and ligand [29,30,31].

In this work, molecular docking and MD simulations (300 K) were performed to investigate the potential of 2-deoxy-D-glucose (2DG—a cyclic molecule), against the inhibition of the main protease of SARS-CoV-2. The results were compared with those from 2-deoxyglucose (2DAG), an acyclic molecule with the same number of carbon atoms, as well as with 2-deoxy-D-ribose (2DR). Furthermore, temperature-dependent MD simulations of the Mpro of nCoV with 2DG was performed to know the inhibition. DFT calculations of 2DG, 2DAG and 2DR were performed to find various energies, the localization of electron density on FMO, and the dipole moment in the gaseous state as well as in water.

## 2. Materials and Methods

### 2.1. Designing of the Ligands

**Figure 1:**
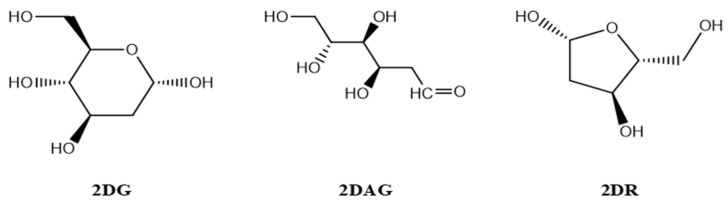
Structures of 2-deoxy-D-ribose ((2DR), 2-deoxy-glucose (2DAG) and 2-deoxy-D-glucose (2DG)).

The structures of the three molecules, namely 2-deoxy-D-ribose (2DR—cyclic form of ribose), 2-deoxy-glucose (2DAG—acyclic form of glucose) and 2-deoxy-D-glucose (2DG—cyclic form of glucose), as shown in Figure 1, were drawn using Chemdraw [32]. The crystal structure of the main protease of the SARS-CoV-2 was taken from the RCSB (PDB-6LU7) and the structure was prepared using ChimeraX (http://www.cgl.ucsf.edu/chimera (accessed on 11 May 2021 and 8 August 2021)). The structure of the protein/receptor/Mpro of nCoV was opened in Chimera and the ligands presented in the pdb were removed. Molecules of solvent in the pdb were deleted. Selenomethionines were replaced by the methionines and charges were assigned. Further, hydrogens were added to justify the valency on the atom in the main protease of nCoV [33,34].

### 2.2. Molecular Docking

Molecular docking is a computational tool used to study the binding of a ligand with a protein/receptor. Effective docking algorithms efficiently explore high-dimensional spaces and employ a scoring system that rates candidate docking. When a ligand binds to the target, docking expresses the results in the form of physical data, that is, binding energy [35,36,37,38,39,40,41]. Many tools for performing molecular docking are available; some of the widely accepted tools are MolDock, iGemdock, ParDock, GOLD, LeDock, etc. In this work, ParDock was used for finding the binding between the molecules and protease [42,43]. It comprises protein–ligand docking techniques based on best fitting of the ligand in the cavity of the protein or receptor. [44,45,46]. The protease was prepared using the chimera, and the addition or deletion of atoms, etc., was performed. The targeted protein was first purged of water, metals, and ligand molecules before being used for molecular docking [34].

### 2.3. Molecular Dynamics (MD) Simulations

MD simulations have been extensively used to investigate the conformation changes, stability and protein–ligand interactions in the complex. It can be performed using various tools. Generally, the MD simulations of the protein–ligand complex are performed by AMBER and GROMACS. In this work, we explored GROMACS, which is used via an online server WEBGRO. It was created by Simlab and made available to academics. (https://simlab.uams.edu/index.php (accessed on 11 May 2021 and 8 August 2021)) Herein, the PDB taken (6lu7) was prepared as per the instruction given on the website, and the topology of the molecules was created by using their associated webserver, named PRODRG (http://davapc1.bioch.dundee.ac.uk/cgi-bin/prodrg (accessed on 11 May 2021 and 8 August 2021)). The data from the docked molecules was taken and fed to the PRODRG server to obtain the zip file, as per the information available. To perform the MD simulations, GROMOS96 43a1, SPC, Triclic were taken as the force field, water model and box type, respectively. Initially, the MD simulations were performed at 300 K to screen the molecules (2DG, 2DAG and 2DR). It provided different trajectories: root mean square deviation (RMSD), root mean square fluctuation (RMSF), radius of gyration (Rg), and hydrogen bonds (HBs). These are the key indicators to visualize the structural features with the movement trajectories. With the help of these trajectories, one can discuss the inhibition of the main protease of SARS-CoV-2 using different promising molecules (2-deoxy-D-ribose, 2-deoxy-glucose and 2-deoxy-D-glucose) [30,47,48,49,50]. Then, the temperature-dependent MD simulations were performed for the Mpro of nCoV in presence of screened molecule using the same conditions [51,52].

### 2.4. Density Functional Theory (DFT)-Based Calculations

DFT calculations used in various fields for estimating the electronic structure of a multibody system. Gaussian 16 is used to perform DFT calculations. DFT with Becke’s three-parameter exchange functional and the Lee–Yang–Paar correlation functional (B3LYP) were applied to perform the geometry optimization and frequency. The basis set 6-311G (d,p) was used in the run the calculations [53,54]. The computations in the solution were carried out using the self-consistent reaction field (SCRF) theory and Tomasi’s polarized continuum model (PCM) [26,27,55,56,57].

## 3. Results

### 3.1. DFT Calculations

**Figure 2:**
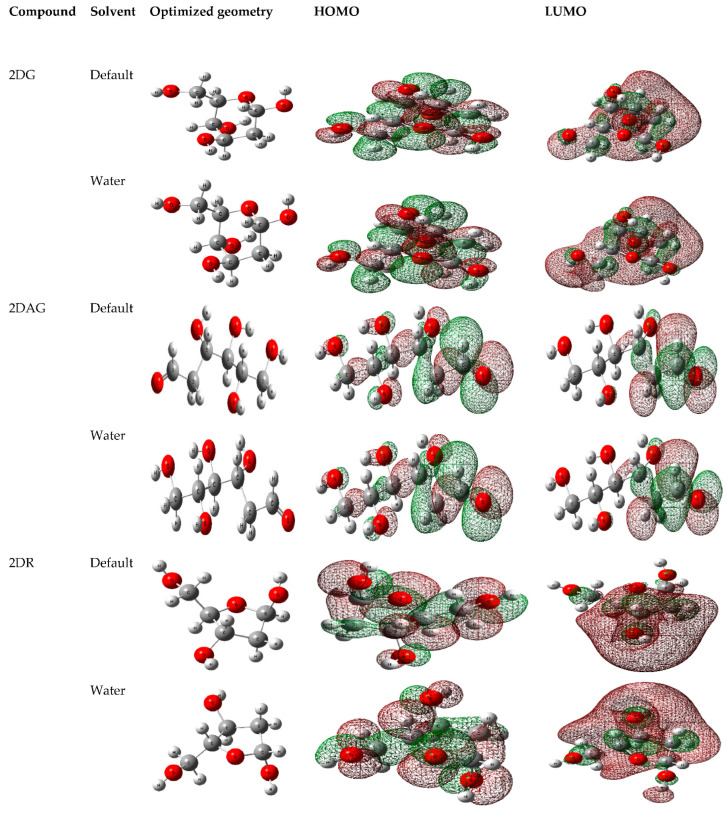
Optimized structures, HOMO and LUMO of 2-deoxy-D-glucose (2DG), 2-deoxy-glucose (2DAG) and 2-deoxy-D-ribose (2DR).

With the help of the DFT calculations, the optimized geometry of each molecule was generated and the localization of electron density on HOMO and LUMO of the 2-deoxy-D-ribose (2DR), 2-deoxy-glucose (2DAG) and 2-deoxy-D-glucose (2DG) were determined in the gaseous state and in water as a solvent, as shown in Figure 2 [58,59,60]. Furthermore, for studying the stability of these molecules, thermodynamic properties such free energy, thermal energy, zero-point energy, optimization energy and the dipole moment in gaseous as well as in aqueous solution at 300 K were calculated.

Furthermore, the DFT calculations provide the thermodynamic parameters calculated of 2DG, 2DAG and 2DR in gaseous and water as well as in water at 300 K (Table 1). It is observed that enthalpy, optimization energy, free energy, zero-point energy and other parameters were slightly higher in water than in the gaseous state. This thermodynamic data provides a relationship to calculate the other important parameters, such as the dipole moment. The dipole moment represents the charge distribution on the molecules. Therefore, it can be used to illustrate the charge density across the molecule. The dipole moment arises due to the difference of electronegativity or more electronegative atoms or functional groups. The greater the variation in electronegativity, the greater the dipole moment will be. The dipole moment of the molecules is slightly higher in water than in the gaseous state. The dipole moment of the molecules in the gaseous phase is based on the polarization or the distribution of electron density [61,62,63,64,65]. The higher value of the dipole moment of 2DAG shows its high solubility in water in comparison of 2DG and 2DR. This indicates that the acyclic molecule shows high solubility, as it has OH groups have less steric hinderance in comparison of the cyclic molecules taken for study.

### 3.2. Molecular Docking

To predict the binding affinity between the main protease of novel coronavirus and the ligand, PARADOCK was used [28,34,45,66]. Using prior information on the initial ligand interaction site, the drug site was observed. From Table 2, it can be observed that 2DAG has the lowest binding energy, followed by 2DG and 2DR, which show −2.88, −2.40 and −2.22 kcal/mol, respectively. The minimum negative binding energy of the 2-DAG molecule defines the stability of the complex bonded with the main protease of nCoV.

**Figure 3:**
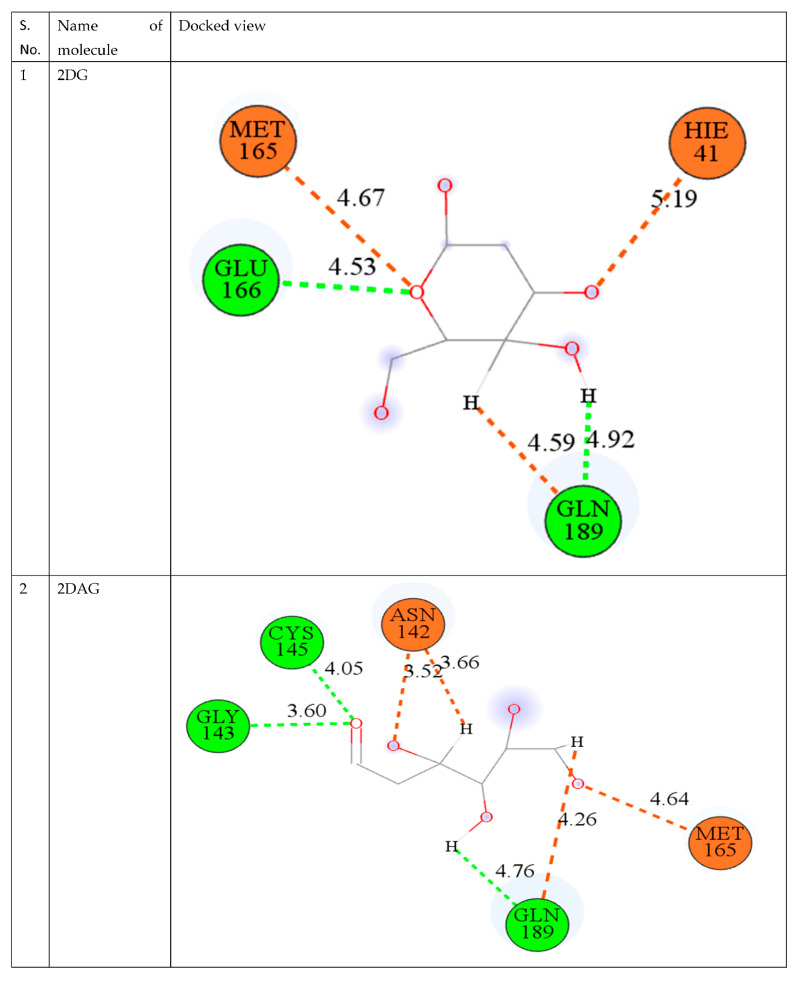

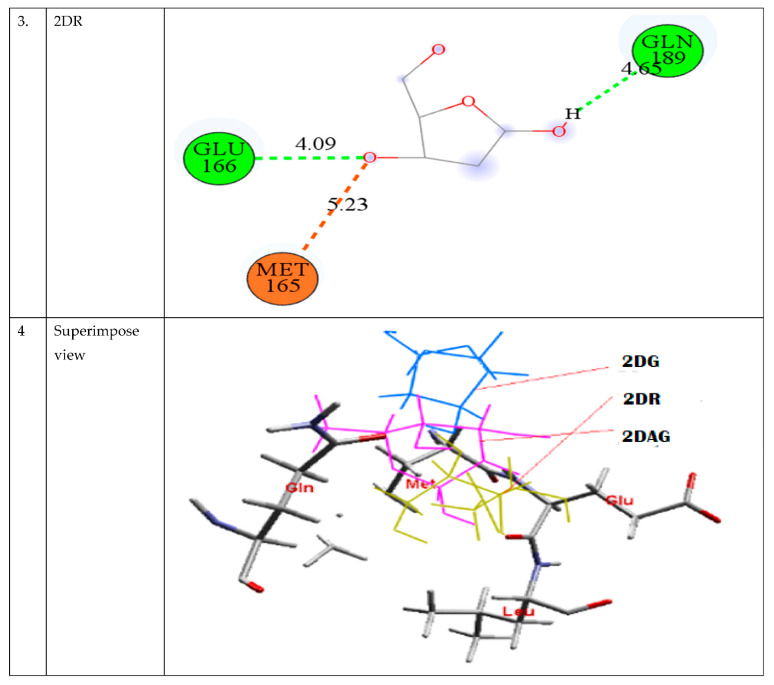
Depiction of the interaction of 2DG, 2DAG, and 2DR with the main protease of novel coronavirus.

The best way to compare many drug compounds is through superposition analysis, which also allows for the prediction of binding sites. In the interaction poses, as shown in Figure 3, it is visible that 2DG and 2DAG have same number of carbon atoms binding and interacts with the same amino acid: GLN 189 (Glutamine) and GLU 166 (Glutamic acid), while for 2DR, one of the interacting amino acid differs. 2DR binds with the histidine amino acid with the oxygen of the carboxyl group and glutamine. As reported, molecular docking provides an idea but not a very reliable tool; therefore, one has to perform MD simulations of the complex of the Mpro of nCoV with the ligand used in molecular docking at 300 K to attain a better understanding of the stability and solubility of the complex formed [67,68,69].

### 3.3. Molecular Dynamics (MD) Simulations

Initially, MD simulations were performed at 300 K to find the most promising candidate against the Mpro of nCoV. After obtaining screened molecules, that is, 2DG, the MD simulations were performed using GROMACS at different temperatures, i.e., 295 K, 310 K and 315 K, to understand the inhibition of the main protease of SARS-CoV-2 in presence of 2DG [53,70,71].

### 3.4. RMSD Trajectories of 2-Deoxy-D-Ribose (2DR), 2-Deoxy-Glucose (2DAG) and 2-Deoxy-D-Glucose (2DG) with the Main Protease of SARS-CoV-2

**Figure 4:**
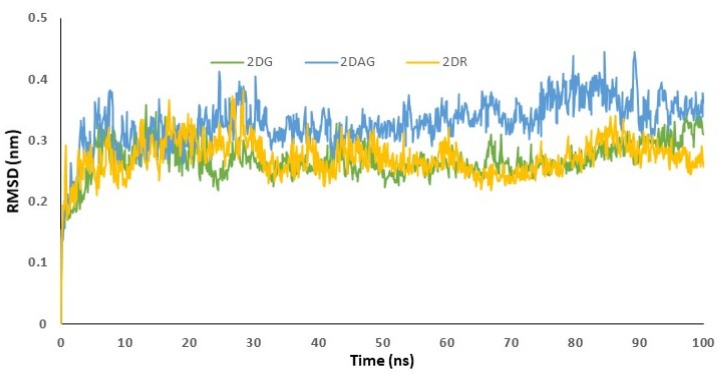
Trajectory of RMSD fit to backbone for the main protease of SARS-CoV-2 with 2-deoxy-D-ribose (2DR), 2-deoxy-glucose (2DAG) and 2-deoxy-D-glucose (2DG).

The data acquired from the docking can be validated using the RMSD trajectory. The interaction of the small molecule with the receptor was depicted by the RMSD trajectories generated by the MD simulations. The structure was assumed to be stiff, and the least-squares approach was used to obtain the global minimum in a timely way. The acceptable range of RMSD values was less than 3 Å. RMSD values are utilized to determine the stability of the Mpro of SARS-CoV-2 in presence of biologically potent molecules by investigating the conformational alterations of the receptor [3,44,72,73]. RMSD determines the average distance between a group of atoms and the influence of ligand on the distance. This means that a change in the conformation of the protein structure is observed. If a decrease in distance is observed, this indicates effective binding and the formation of a stable complex. Figure 4 is used to understand the RMSD of the complex, when 2-deoxy-D-ribose (2DR), 2-deoxy-glucose (2DAG) and 2-deoxy-D-glucose (2DG) fit in the cavity of the protease. Herein, the RMSD values for the complex of 2DAG fluctuate and are high. Therefore, the 2DAG binds less effectively with the Mpro of nCoV. The RMSD values for the complexes formed between the Mpro of nCoV and 2DG/2DR fall within the acceptable range and show effective binding or the formation of stable complexes at 300 K. 2DG and 2DR can be considered to be promising inhibitors.

**Figure 5:**
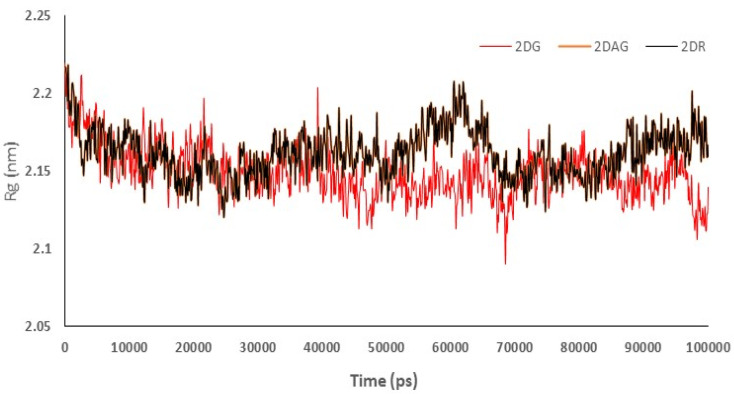
Trajectory of radius of gyration for the main protease of SARS-CoV-2 with 2-deoxy-D-ribose (2DR), 2-deoxy-glucose (2DAG) and 2-deoxy-D-glucose (2DG).

Radius of gyration (Rg) was used to understand the compactness of the main protease of nCoV in presence of 2DG/2DAG/2DR. It offers information about the packing of the secondary structure in three-dimensional structures. Compactness is defined as the ratio of the accessible surface area to the surface area of the ideal sphere of the same volume. Lower values of Rg indicate more a compact structure of the protein. Therefore, the trajectory can be used to investigate the effect of the molecules taken for the change in the compactness of the Mpro of nCoV. It can be seen that 2DG has the maximum compactness of the structure due to minimum Rg values, as shown in Figure 5. From the trajectories we can conclude that 2DAG and 2DR show a high fluctuation in compactness of structure, indicating less stability of the complex, while in the case of 2DG, the values are more acceptable and show less fluctuation (Figure 5).

**Figure 6:**
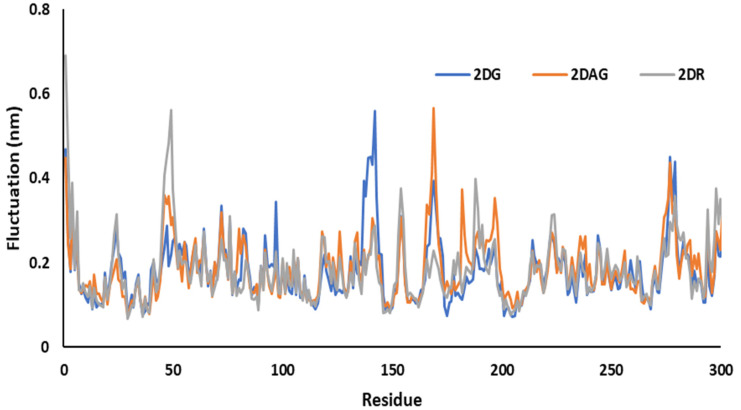
Trajectory of RMSF for the main protease of SARS-CoV-2 with 2-deoxy-D-ribose (2DR), 2-deoxy-glucose (2DAG) and 2-deoxy-D-glucose (2DG).

MD simulations were used to investigate the RMSF of the main protease of novel coronavirus with molecules at 100 ns time period. The flexibility of each amino acid or the residue in the complex was determined using RMSF plots. The resulting structural variations were minimal. It has been determined that certain inhibitors may interact with target protein residues. It studies the change or fluctuation of the position of the amino acids of the Mpro of nCoV in the presence of the ligand. For 2DG, 2DAG and 2DR, the maximum fluctuation was observed around 140, 165 and 50, respectively. 2DG had the minimum fluctuation in comparison with 2DAG and 2DR, and could be a better inhibitor of the Mpro of nCoV (Figure 6).

**Figure 7:**
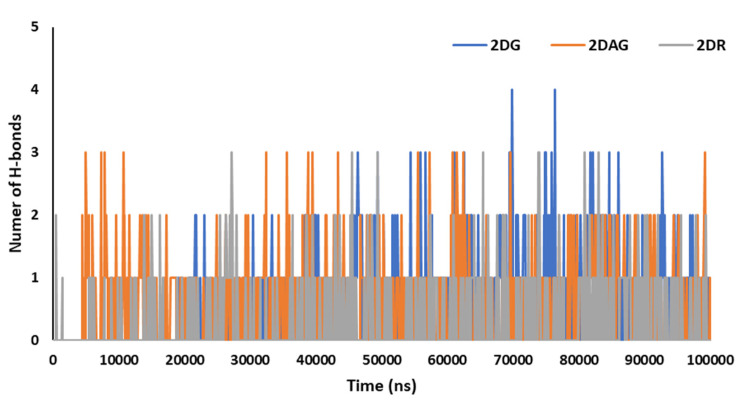
Trajectory of hydrogen bonds for the main protease of SARS-CoV-2 with 2-deoxy-D-ribose (2DR), 2-deoxy-glucose (2DAG) and 2-deoxy-D-glucose (2DG).

Hydrogen bonds are significant for studying the binding affinity and structural stability of the complex produced between the Mpro of nCoV and the ligand. The percentage occupancy can be computed via HBs formation and the angle during simulation time. The number of hydrogen bonds induced by the Mpro of SARS-CoV-2 with the D-ribose (2DR), 2-deoxy-glucose (2DAG) and 2-deoxy-D-glucose (2DG) can be seen in Figure 7. It can be seen that the maximum number of hydrogen bonds between the Mpro and 2DG, 2DAG and DR are four, three and three, respectively. 2DG forms a greater number of hydrogen bonds with the Mpro than 2DAG or 2DR. Therefore, 2DG may be considered as a better and more promising candidate for the inhibition of the main protease of SARS-CoV-2.

Based on the MD simulation results obtained at 300 K for the Mpro of nCoV with 2DG, DAG and 2DR, 2DG was found to be the most prominent inhibitor. Therefore, we thought to perform the MD simulations of the Mpro of nCoV with 2DG at various temperatures, that is, 295, 310 and 325 K, to investigate the effect of temperature.

### 3.5. Temperature-Dependent MD Simulations for Mrpo of nCoV with 2DG

**Figure 8:**
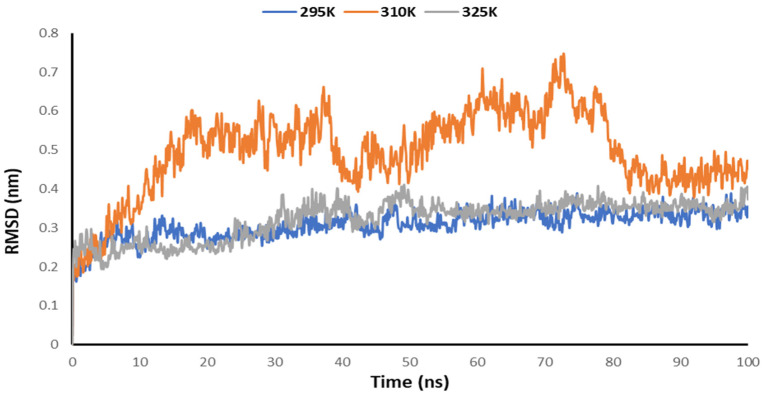
Trajectory of RMSD for the main protease of SARS-CoV-2 with 2DG at 295, 310 and 315 K.

Temperature-dependent MD simulations were conducted for the complex of the Mrpo of SARS-CoV-2 with 2DG to understand the effect of temperature on its activity. From the MD simulations, no regular pattern was observed upon increasing the temperature with the help of Rg, RMSD, RMSF and the number of hydrogen bonds. Figure 8 explains the RMSD of the Mpro of nCoV in the presence of 2DG at various temperatures to understand the deviation of the backbone of the main protease of novel coronavirus. Although, no regular pattern was observed, the deviation of the atomic coordinates was at a minimum, at 295 K. This means that the binding is effective and forms a stable complex.

**Figure 9:**
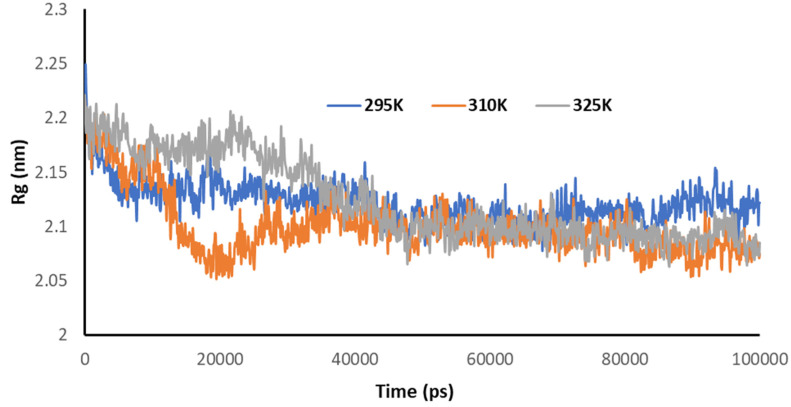
Trajectory of Rg for the main protease of SARS-CoV-2 with 2DG at 295, 310 and 315 K.

Figure 9 explains the radius of gyration of the Mpro of nCoV in the presence of 2DG at different temperatures. It can be seen that the structural conformation was found at a minimum at 295 K, indicating the maximum stability of the complex or the effective/maximum binding. This means that increasing the temperature decreased the binding of 2DG with the Mpro of nCoV.

**Figure 10:**
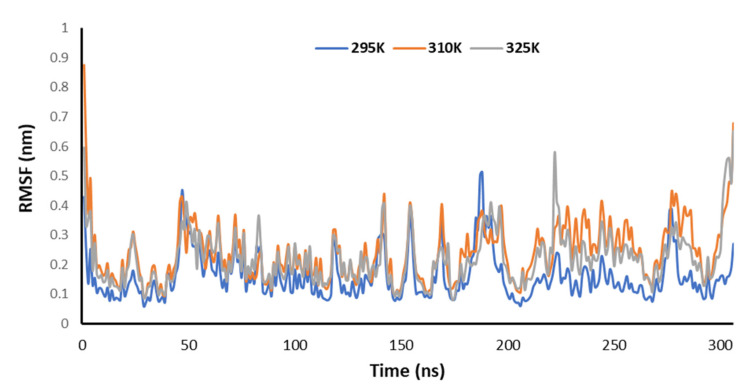
Trajectory of RMSF for the main protease of SARS-CoV-2 with 2DG at 295, 310 and 315 K.

Figure 10 explains the RMSF of the Mpro of nCoV in the presence of 2DG at various temperatures (295, 310 and 315 K) to understand the fluctuation. The lowest fluctuations were observed at 295 K, meaning that the binding is effective and forms a stable complex at 295 K.

**Figure 11:**
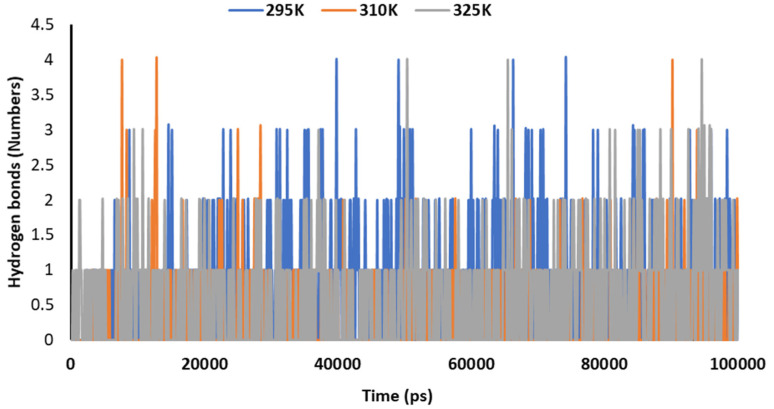
Trajectory of hydrogen bonds for the main protease of SARS-CoV-2 with 2DG at 295, 310 and 315 K.

Figure 11 explains the hydrogen bonding in the complex of 2DG with the Mpro of nCoV at various temperatures (295, 310 and 315 K) to understand the stability. The maximum number of hydrogen bonds were formed at 295 K, indicating effective binding and maximum stability.

## 4. Discussion

The novel coronavirus and its strains have threatened humanity. Therefore, there is a need to develop an efficient drug like candidate to save lives. 2DG was permitted for emergency use to control infection of SARS-CoV-2 in lung host cells during the second wave of COVID-19 in India. According to recent reports, clinical trial data show that 2DG helps to hasten recovery of patients hospitalized with COVID-19 and reduces their dependence on supplemental oxygen. Generally, infected cells have the ability to consume glucose and therefore take huge amounts of 2DG. Thus, 2DG reported to hampers the growth of the cells. Its selective accumulation in virally infected cells makes this drug promising. 2DG can have immense benefit for people suffering from COVID-19. 2DG is available in powder form in a small packet, which can be taken orally by dissolving it in water. It accumulates in the virus-infected cells by targeting metabolism. Herein, 2DG and other mimics such as 2DAG and 2DR were used for this study to target the Mpro of SARS-CoV-2. In this work, molecular docking and MD simulations were performed to investigate and compare the potential of 2DG, 2DAG and 2DR for the inhibition of the main protease of SARS-CoV-2. Based on the results obtained by molecular docking, 2DAG was found to have the lowest binding energy compared with 2 DG and 2 DR, so that it might be considered a more promising candidate. However, as we know, the results obtained by molecular docking are not very reliable and only give an idea. Therefore, MD simulations of the Mpro of nCoV with these molecules was performed at 300 K. 2DG was found to be a better inhibitor of the Mpro of nCoV than 2DAG and 2DR based on the different trajectories obtained by MD simulations at 300 K. Furthermore, the temperature-dependent MD simulation was performed at temperatures of 295, 310 and 315 K of the Mpro of nCoV with 2DG to study the effect of temperature on the inhibition of protease. The maximum inhibition of the Mpro of nCoV was observed at 295 K. Furthermore, DFT calculations of 2DG, 2-DAG and 2-DR were performed to determine the different energies, the localization of electron density on FMO, and the dipole moment of the compounds in a gaseous state as well as in water at 300 K. The molecules in the gaseous phase produces more polarization and resulting more dipole moment, while in the liquid phase the value is comparatively higher than in the condensed. The dipole moment explained the solubility of the designed molecules in water.

## 5. Conclusions

In the present work, molecular docking was performed to find the potential of 2DG for the inhibition of the Mpro of nCoV and compare it’s potential with 2DR and 2DAG. The binding energy of 2DG, 2DR and 2DAG against the Mpro of nCoV was −2.40, −2.22 and −2.88 kcal/mol, respectively. These results provide only an idea. For a better understanding, therefore, MD simulations of the Mpro of nCoV with 2DG, 2DR and 2DAG were performed at 300 K. Based on the results, 2DG was found to be a better inhibitor for the Mpro of nCoV than 2DAG and 2DR. Furthermore, the temperature-dependent MD simulations of the Mpro of nCoV with 2DG were performed at 295, 310 and 315 K. It was observed that effective binding occurs at 295 K. DFT calculations of 2DG, 2DAG and 2DR were also performed to find the various energy, the FMO and the dipole moment in the gaseous state as well as in water at 200 K to know their stability and solubility.

## Figures and Tables

**Table 1:** Various thermodynamic parameters of 2DG, 2DAG and 2DR.

	Solvent	Sum of Electronic and Zero-Point Energies	Sum of Electronic and Thermal Energies	Sum of Electronic and Thermal Enthalpies	Sum of Electronic and Thermal Free Energies	Optimization Energy	Dipole Moment
**2DG**	Default	−611.93	−611.92	−611.91	−611.97	−612.12	3.4
	Water	−611.95	−611.94	−611.94	−611.98	−612.14	4.7
**2DAG**	Default	−611.93	−611.92	−611.91	−611.97	−612.12	8.17
	Water	−611.95	−611.94	−611.94	−611.99	−612.14	10.04
**2DR**	Default	−497.40	−497.39	−497.39	−497.44	−497.56	2.35
	Water	−497.42	−497.41	−497.45	−497.45	−497.58	3.1

**Table 2:** Binding energy of the molecules.

S. No.	CMPD	Binding Energy (kcal/mol)
1.	2DG	−2.40
2.	2DR	−2.22
3.	2DAG	−2.88
